# Women’s Participation in Pap Smear Screening in a Developing Country: Evidence for Improving Health Systems

**DOI:** 10.3389/fonc.2021.642841

**Published:** 2021-06-15

**Authors:** Carolina Santamaría-Ulloa, Ileana Quirós-Rojas, Melina Montero-López, Hazel Quesada-Leitón

**Affiliations:** ^1^ Instituto de Investigaciones en Salud, Universidad de Costa Rica, San Jose, Costa Rica; ^2^ Coordinacion Tecnica del Cancer, Caja Costarricense del Seguro Social, San Jose, Costa Rica; ^3^ Escuela de Estadística, Universidad de Costa Rica, San Jose, Costa Rica

**Keywords:** Papanicolaou, prevention, cervical cancer, screening, women’s health

## Abstract

**Introduction:**

Every year about 83,000 women are diagnosed with cervical cancer in the Americas. Latin America and the Caribbean (LAC) has one of the highest incidence and mortality rates from cervical cancer in the world. Although incidence has decreased by half in the last 30 years, cervical cancer remains a public health concern. The detection of precursor lesions through Papanicolaou (Pap) smear remains a critical tool in the context of prevention in Costa Rica and many other LAC countries.

**Objective:**

To determine predictors of participation in Pap smear screening among Costa Rican women, with a special focus on women who have never had a Pap smear or have had a smear 5 or more years ago.

**Methods:**

The data source for this study is the Costa Rican Households National Survey conducted in 2014. This survey is representative at the national, urban/rural zone, and administrative region level. A subsample of women aged 20 to 69 years who responded to the survey’s Papanicolaou Module were included in this study (n = 11,709). Statistical analyses were conducted in R-Studio. Statistical significance level was set at 5%. Two multinomial regression models were estimated. The first model aimed to explain the five different categories of cytology use, which were defined according to the last time women had a Pap smear. The second model aimed to explain the five different categories of reasons why women had never had a Pap smear. Both models controlled for age, educational attainment, and marital status.

**Results:**

Young women with high educational attainment were more likely to have never had a cytology. Women with a lower educational attainment, married, or in domestic relationship and of older age had greater odds of having had a cytology 5 or more years ago. Each year increment in age was significantly associated with an increase in the odds of never having a Pap smear because of health care access issues or because of cultural reasons as compared to not having an active sexual life.

**Conclusions:**

Findings can inform public policy targeted to higher risk female populations where access to health services can be improved.

## Introduction

Cervical cancer impacts a substantial number of women. Every year about 83,000 women are diagnosed with cervical cancer in the Americas, and about 36,000 die of this condition. Latin America and the Caribbean (LAC) has one of the highest incidence and mortality rates from cervical cancer in the world (1). In the Americas, cervical cancer is the most common cause of death in eleven countries, and the second most common cause of death in another 12 countries (2). In many Latin American cities, cervical cancer incidence is even higher than the world average (3). In Costa Rica, an LAC country, cervical cancer is the third most incident female cancer. In 2011, cervical cancer adjusted incidence rate was 27 per 100.000 women. In 2013, a total of 133 cervical cancer deaths occurred in the country, 38% of them in women younger than 50 years (4).

Morbidity and mortality are determined and influenced by life conditions that occur in different spaces of social reproduction, known as the social determinants of health. Social determinants include the economic and political structure, the cultural organization, the distribution of goods and services, gender inequalities, and individual biological conditions (5). Social determinants are embedded in the population health outcomes. Even among the biological conditions that are expressed at the individual level, the resources an individual has for self-care and for seeking health care, are socially determined.

Social determinants explain how women with disadvantaged life conditions, such as women in developing countries, are at a greater risk of cervical cancer. In this context, the Pan American Health Organization recommends interventions to reduce access barriers to individuals who live under adverse social and economic circumstances (2).

Among the individual and biological determinants of cervical cancer are the persistent infections by the oncogenic types of human papillomavirus (HPV), which are the most common viral infections of the reproductive system (6). According to WHO (7) an integral control of cervical cancer should embrace 1) primary prevention, which includes sexual education programs, as well as vaccination against HPV; 2) secondary prevention, which includes screening strategies such as cytology, as well as treatment of precancerous lesions; and 3) tertiary prevention, which includes diagnosis and treatment of invasive cancer.

To warrant the effectiveness of strategies aimed at controlling cervical cancer, it is necessary for women to gain access to a health system with the capacity of acting at the three levels of care, from sexual education, to diagnosis and follow-up when needed. Expanding prevention programs, such as vaccination, screening, and treatment of precursor lesions has been challenging in Latin America (8). Barriers to access are central in this context, since they put women into a greater risk of disease and death of cervical cancer. Bridging inequalities in women life conditions is the greatest challenge that Latin American countries face to control cervical cancer.

Cytology-based (Pap smear) population screening programs for early detection have been implemented in LAC for the last four decades (9). Pap smear programs face limitations and challenges, but remain the most widely used screening programs in the region. The target population for these screening programs has not been universally defined across LAC countries in terms of screening age and periodicity. In most countries, screening starts at age 25 years (ranging from 15 to 35 years) and ends at age 59 years (ranging from 49 to 69 years) (10).

Costa Rica’s universal health care system was implemented in 1961. The Costa Rican Social Security Fund (CCSS, for its Spanish acronym) is the national entity responsible for health care provision. In order to decrease and control the prevalence of cervical cancer, during the 1960s the CCSS integrated vaginal cytology into family planning appointments. During the 1970s, actions were taken to strengthen prevention and treatment of cervical cancer, such as increasing infrastructure, building professional capacity and obtaining medications and equipment (11). In 2007, national guidelines were published with the aim of providing women a better access to cervical cancer prevention and standardizing health care at the three levels of care. According to the guidelines currently in place, Pap smear must be conducted every 2 years on women 20 years or older who ever had coital intercourse, regardless of their insurance status (12).

Besides Pap smear screening, Costa Rica has started a pilot-phase introduction of a molecular test for HPV screening in women aged 30 to 64 years (13) and has also taken part in the Comparative Study on one- and two-dose HPV vaccines (ESCUDDO, for its Spanish acronym), which assesses if the one-dose HPV vaccine has similar efficacy to the two-dose vaccine. Vaccination itself does not completely eliminate the probability of an HPV infection. Screening, diagnosis, and treatment remain important components of any country’s program, and therefore, the identification of barriers to participation in national programs is highly relevant.

Individual and structural level factors determine health outcomes, by interacting with each other (14). Women’s participation in Pap smear screening programs is mediated by both individual and structural level factors. Some individual factors, such as health behavior, cultural beliefs, or education, may be modifiable. Some structural level factors, such as socioeconomic status, cannot be addressed by screening programs. Nonetheless, other structural factors related to access to the health care system (15), or to gender inequalities, may be modifiable by taking actions based on evidence.

Although incidence and mortality have considerably decreased in the last 30 years, cervical cancer remains a public health concern. Preventable mortality is still occurring (16). The identification of deprived or higher risk population groups to be targeted is critical. The detection of precursor lesions through Pap smear remains a very important tool in Costa Rica and many other LAC developing countries (11). The objective of the present study is to determine predictors of participation in Pap smear screening among Costa Rican women, with a special focus on women who have never had a Pap smear or have had a smear 5 or more years ago. Study findings will inform the identification of groups that can be targeted to attain higher rates of participation in Pap smear screening programs.

## Methods

The data source for this study is the Costa Rican Households National Survey conducted in 2014. This survey is representative at the national, urban/rural zone, and administrative region level. It included a total of 13,400 households. A subsample of women aged 20 to 69 years who answered the Papanicolaou smear Module (Pap smear Module) of the survey were included in this study (n = 11,709). Although the Pap smear Module was applied to women 18 years or older, the national guideline for cervical cancer refers to women 20 years or older, and this is the population subgroup included in this study.

### Estimated Weights

The Households National Survey total sample contains a weight estimation for each observation, which allows for national level inferences. This study was based on the Pap smear Module, which was applied to a subgroup of the sample defined by age and gender of the participants. To maintain a nationally representative sample, for each observation included in this study, a weight factor was estimated as the result of dividing the original observation weight by the average of all of the weights from the total sample. Applying this constant factor to the observations was important because it allowed to keep representativity of the characteristics on which the sample estimation was based, without expanding the number of observations (17).

### Variables

The variables under study were: time elapsed since the last Pap smear, reasons to have never had a Pap smear, and sociodemographic characteristics.

Time elapsed since the last Pap smear was classified into the following categories: 1) Less than 1 year, 2) 1 to less than 2 years, 3) 2 to 3 years, 4) 3 to less than 5 years, and 5) 5 or more years.

Reasons to have never had a Pap smear were classified as follows: 1) Cultural: barriers from the husband or partner; out of shame or fear; carelessness; intentional postponement; lack of time; considering it not important, considering it not necessary. 2) Non-active sex life: currently does not have an active sex life; has never had an active sex life. 3) Economic difficulties: cannot afford the screening; does not have health insurance. 4) Health system access issues: difficulty obtaining an appointment. 5) Other reasons: geographical barriers; work commitments; lack of a care provider for children or elderly; other.

Sociodemographic variables included age, educational attainment, and marital status. Age was a continuous variable. Educational attainment was categorized as 1) Incomplete primary, 2) Complete primary, 3) Incomplete High school, 4) Complete high school and 5) College or higher. Marital status was categorized as 1) In partnership: married or in domestic relationship, and 2) Not in partnership: single, separated or divorced.

### Multinomial Regression Model

Statistical analyses were conducted in R-Studio. The following libraries were used: dplyr (18), MASS (19), nnet (19), survey (20). The significance level was set at 5%.

Two multinomial regression models were estimated. The first model aimed to explain the five different categories of cytology use, which were defined according to the last time women had a Pap smear. The second model aimed to explain the five different categories of reasons why women had never had a Pap smear. Both estimated models controlled for age, educational attainment, and marital status.

For each independent variable in the multinomial regression model, an associated parameter exists. Significance was evaluated by the Wald test as described by Pont (21) and by Agresti (22), which allowed evaluation of whether variables included in the model were significantly different from zero, that is, if the variable helped to describe the phenomena under study. A general principle of statistical inference is that the larger the sample size, the greater the control of random error and the greater the power of the hypothesis test to detect differences of any magnitude given they exist. Therefore, the rejection of a null hypothesis greatly depends on the sample size. A large sample size will almost always allow for rejection of the null hypothesis, whereas a small sample size will hardly attain statistical significance (23). Given the fact that statistical inference was being conducted in this study, weights were estimated as aforementioned, without expanding the number of observations, in order to avoid attaining statistical significance as an artifact of a large sample size (17).

The multinomial regression model is described by the following equation:

$$P\left(Y=j|X\right)=\frac{e\left(X_{j}\beta_{j}\right)}{1+\sum_{i=1}^{j}e\left(X_{j}\beta_{j}\right)}\;;j=1,2,3$$

Where:

J = categories of the dependent variable 

Y = dependent variable

X = independent variables

The dependent variable *Y* of the two estimated models was “Time elapsed since the last Pap smear”, and “Reasons to have never had a Pap smear”, respectively; with their corresponding *J* categories. In the first model, 1 to less than 2 years since the last Pap smear was the *J* reference category. In the second model, not having an active sexual life was the *J* reference category. For both models the *X* independent variables were: age, educational attainment, and marital status.

## Results

The main characteristics of the population under study are presentsed in **Table 1**. Twenty-eight percent of women were aged 20 to 29 years, 55% were married or in a domestic relationship, 37% had either complete or incomplete primary education, and 70% had their last vaginal cytology less than 2 years ago, which is the screening periodicity established in the country’s national guidelines. Because of women’s lack of or low participation in the Pap smear screening program, this study focused on two populations: women who have never had a Pap smear, and women who have had a smear 5 or more years ago.

**Table 1:** Descriptive information of the study population.

Variable	Frequencies		Last time women had a Pap smear	
	Absolute	Relative	Never	5+ years ago
**Age**				
20–29	3,343	27.8	72.4	11.5
30–39	2,789	23.2	13.7	23.0
40–49	2,532	21.0	6.0	24.8
50–59	2,525	21.0	5.8	29.4
60–64	850	7.1	2.1	11.3
Total	12,039	100.0	100.0	100.0
**Marital status**				
Married/in domestic relationship	6,640	55.2	15.0	54.0
Not in partnership/single	5,399	44.8	85.0	46.0
Total	12,039	100.0	100.0	100.0
**Educational attainment**				
Incomplete primary	1,481	12.3	9.8	20.0
Complete primary	2,917	24.3	11.5	30.0
Incomplete High school	2,274	18.9	16.3	23.3
Complete High school	2,150	17.9	22.6	11.3
College degree or higher	3,183	26.5	39.8	15.4
Total	12,005	100.0	100.0	100.0
**Last time women had a Pap smear**				
Never	1,386	11.7	-	-
Less than 1 year ago	5,251	44.2	-	-
1 to < 2 years ago	3,002	25.3	-	-
2 to < 3 years ago	1,211	10.2	-	-
3 to < 5 years ago	529	4.5	-	-
5+ years ago	496	4.2	-	-
Total	11,876	100.0	-	-

Women who had never had a cytology were younger than the total population under study (72% were aged 20-29 years); a higher proportion of them were single or were not in domestic relationship (85%); and had a higher educational attainment (62% had a high school or college degree), as compared to the total population. Women who had had a Pap smear 5 or more years ago were older than the total population under study (41% were aged 50+ years); similar to the total population, they were almost equally distributed between married and single (54% were married or in domestic relationship); but had a lower educational attainment than the total female population (50% had complete primary school or a lower educational attainment) (**Table 1**).

An educational attainment profile of the female population with null or low participation in the Pap smear screening program yielded interesting results. On one hand, as educational attainment improved, there was an increase in the proportion of women who never had a Pap smear. Among those with incomplete primary school (n=1464), only 9% had never had a screening, whereas among women with a college degree or higher (n=3118), 18% had never had a Pap smear. On the other hand, as educational attainment improved, there was a decrease in the proportion of being married and never having had a Pap smear. Among those with incomplete primary school (n=1464), 34% had never had a screening, whereas among women with a college degree or higher (n=3118), 18% had never had a Pap smear (**Table 2**).

**Table 2:** Last time women had a Pap smear, by marital status and educational attainment.

Educational attainment	Last time women had a Pap smear	Total	Marital status	
			Married/In domestic relationship	Not in partnership/Single
Incomplete primary	Never	136	33.82	66,18
	Less than 1 year ago	597	64.15	35.85
	1 to < 2 years ago	353	63.17	36.83
	2 to < 3 years ago	183	63.39	36.61
	3 to < 5 years ago	97	47.42	52.58
	5+ years ago	98	56.12	43.88
Complete primary	Never	159	28.30	71.70
	Less than 1 year ago	1323	64.78	35.22
	1 to < 2 years ago	829	69.84	30.16
	2 to < 3 years ago	304	67.43	32.57
	3 to < 5 years ago	133	69.17	30.83
	5+ years ago	147	55.10	44.90
Incomplete High school	Never	226	17.26	82.74
	Less than 1 year ago	1012	60.57	39.43
	1 to < 2 years ago	556	58.09	41.91
	2 to < 3 years ago	221	64.25	35.75
	3 to < 5 years ago	109	55.05	44.95
	5+ years ago	114	50.00	50.00
Complete High school	Never	313	13.74	86.26
	Less than 1 year ago	918	57.41	42.59
	1 to < 2 years ago	503	58.25	41.75
	2 to < 3 years ago	244	60.25	39.75
	3 to < 5 years ago	93	65.59	34.41
	5+ years ago	56	60.71	39.29
College degree or higher	Never	550	6.36	93.64
	Less than 1 year ago	1382	57.96	42.04
	1 to < 2 years ago	753	56.97	43.03
	2 to < 3 years ago	258	53.10	46.90
	3 to < 5 years ago	99	53.54	46.46
	5+ years ago	76	50.00	50.00

Another finding was that as educational attainment improved, there was a decrease in the proportion of women who had a Pap smear 5 or more years ago. Among those with incomplete primary school, 7% (98 out of 1464) had never had a screening; whereas only 2% (76 out of 3118) of women with a college degree or higher had a Pap smear 5 or more years ago. No clear educational gradient was found for married women who had a screening 5 or more years ago (56% for incomplete primary school as compared to 50% for women who attained college or a higher education level) (**Table 2**).

Multinomial regression results show that educational attainment was significantly associated with participation in Pap smear screening. Using 1 to less than 2 years as the reference category, a dose-response relationship between time elapsed since the last Pap smear and educational attainment was evident. Women with incomplete primary were significantly more likely of having never had a Pap smear (OR: 1.51; p< 0.01), or having had it 5 years ago or longer (OR: 2.27; p<0.01). Age also increased the odd of having a Pap smear 5 years ago or longer. Each ten-year increment of age was associated with a 23.6% increase in the odd of having a Pap smear 5 or 3 more years ago (OR = 1.02; p < 0.01) (**Table 3**).

**Table 3:** Last time women had a Pap smear, by age, educational attainment, and marital status.

Last time women had a Pap smear	Variable	OR	Associated probability	
Never^1^	Age	0.91	<0.01	*
	Incomplete primary^2^	1.51	<0.01	*
	Complete primary	0.64	<0.01	*
	Incomplete High school	0.64	<0.01	*
	Complete High school	0.81	0.04	*
Less than 1 year ago^1^	Age	1.00	0.07	
	Incomplete primary^2^	0.93	0.41	
	Complete primary	0.88	0.04	*
	Incomplete High school	0.99	0.87	
	Complete High school	0.99	0.86	
2 to < 3 years ago^1^	Age	1.00	0.28	*
	Incomplete primary^2^	1.54	0.00	*
	Complete primary	1.08	0.46	
	Incomplete High school	1.16	0.18	
	Complete High school	1.41	<0.01	*
3 to< 5 years ago^1^	Age	1.00	0.69	
	Incomplete primary^2^	1.97	<0.01	*
	Complete primary	1.19	0.24	
	Incomplete High school	1.47	0.01	*
	Complete High school	1.39	0.03	*
5+ years ago^1^	Age	1.02	<0.01	*
	Incomplete primary^2^	2.27	<0.01	*
	Complete primary	1.58	<0.01	*
	Incomplete High school	1.99	<0.01	*
	Complete High school	1.10	0.62	

^1^1 to < 2 years ago is the reference category.

^2^Women with college degree or higher, are the reference category. *P < 0.05.

Multinomial regression model results for reasons why women have never had a Pap smear screening are presented in **Table 4**. Our results show that each year increment in age was associated with a 7% increase (OR=1.07; p< 0.01) in the odds of never having a Pap smear because of health care system access issues (difficulty getting an appointment), as compared to not having an active sexual life. Similarly, each year increment in age was associated with a 5% increase (OR = 1.05; p = 0.02) in the odds of never having a Pap smear because of cultural reasons (husband or partner prevents; shame or fear; carelessness; woman has been postponing; lack of time; considering it not important, or considering it not necessary), as compared to not having an active sexual life.

**Table 4:** Reasons why women have never had a Pap smear screening.

Reasons	Variable	OR	Associated probability	
Cultural/personal ^1^	Age	1.05	0.02	*
	Incomplete primary^2^	3.04	0.03	*
	Complete primary	0.63	0.13	
	Incomplete High school	1.94	0.01	*
	Complete High school	1.09	0.69	
	Not union/Single	0.05	<0.01	*
Economic difficulties^1^	Age	1.01	0.49	
	Incomplete primary^2^	15.68	<0.01	*
	Complete primary	2.90	0.02	*
	Incomplete High school	6.07	<0.01	*
	Complete High school	4.74	<0.01	*
	Not union/Single	0.06	1.00	
Health system^1^	Age	1.07	<0.01	*
	Incomplete primary^2^	0.20	0.34	
	Complete primary	0.46	0.30	
	Incomplete High school	0.65	0.60	
	Complete High school	0.56	0.41	
	Not union/Single	0.03	1.00	
Other reasons^1^	Age	1.08	<0.01	*
	Incomplete primary^2^	0.83	0.79	
	Complete primary	0.24	0.00	*
	Incomplete High school	0.55	0.27	
	Complete High school	1.22	0.56	
	Not in partnership/Single	0.04	<0.01	

^1^Women who do not have an active sex life are the reference category.

^2^Women who have college or more, are the reference category. *P < 0.05.

As also shown in **Table 4**, women with a lower educational attainment had increased odds of never having a Pap smear because of economic reasons, as compared to not having an active sexual life. Compared to women with a college degree or higher, the odds of never having a Pap smear were higher among women with incomplete primary (OR = 15.68, p < 0.01), complete primary (OR = 2.90, p = 0.02), incomplete high school (OR = 6.07; p < 0.01), and complete high school (OR = 4.74; p < 0.01).

## Discussion

Women’s participation in screening programs remains relevant in the LAC region, where cervical cancer incidence and mortality are higher than in other regions in the world, except Africa. This study identified two female populations that can be targeted to attain higher participation in screening programs.

A first identified target population was young, single women, with a high educational attainment; who were more likely to have never had a cytology in Costa Rica. A second target population was older, married women, with a low educational attainment; who were more likely to have had their last cytology 5 or more years ago.

Although age is clearly a non-modifiable individual factor, it is very important for screening programs to determine to whom they should direct their actions, with educational or other strategies. Our results for women younger than 30 years who have never had a Pap smear or women aged 50 years or older who had their last screening 5 or more or more years ago should help health authorities define age-specific strategies. The study findings regarding women with low participation in screening are consistent with another study conducted in Costa Rica which concluded that women aged 50 to 59 years should be a target group for screening, given the evidence that cervical cancer incidence has been disproportionally affecting this age group in the last decades (24). Age is also a predictor of participation in screening programs in other Latin American countries, such as Mexico, Brazil, and Colombia (25–27).

Our findings regarding education are similar to what has been reported in other studies where educational attainment is an important determinant of participation in early detection programs. In a study conducted in Mexico by Lazcano-Ponce and colleagues (28), both educational attainment and knowledge on the usefulness of Papanicolaou, were identified as the main conditionings associated with women participation in early detection programs. Other studies in Brazil, Peru, and Colombia have also previously described an association between education and participation in Pap smear screening programs (25, 27, 29, 30).

Costa Rica has a high literacy rate. Population-level literacy rates reach 98.5% (31). On average, the Organization for Economic Co-operation and Development (OECD) countries expend 3.1% of their Gross Domestic Product (GDP) on public educational institutions, whereas Costa Rica’s total expenditure reaches 4% of GDP, higher than the United Kingdom, Switzerland, and the United States of America (32). Screening programs should customize their educational strategies to the level of educational attainment of the population Younger women with a high educational attainment need to be encouraged to start participating in screening programs, and should receive information that is differentiated from the information delivered to older women with a lower educational attainment who need to be encouraged to continue their screenings. Knowledge on health-related issues and, more specifically, on awareness and knowledge on the purpose of the test should be population-specific in order to attain better results.

Behavioral change models, especially those related to health behaviors, state that more than information delivery is needed to attain health-related behavioral change (33). Because social, cognitive and emotional factors interact with each other to produce behavioral change at the population level, health interventions should take into consideration a complete profile of the female population to be targeted in order to attain higher Pap smear screening participation rates.

Married women or women in a domestic relationship, when aged 50 or older, were found to have a low participation in screening in Costa Rica. Studies in Latin American countries such as Peru and Mexico have found a significant association between marital status and Pap smear screening practice (26, 27, 29). Marital status has been previously described as a barrier to Pap smear screening through gender inequality, specifically expressed when men do not allow their wives or partners to have a pelvic examination if conducted by male health care personnel (15). A study conducted in Yucatan, Mexico identified gender roles associated with marriage as an indirect barrier to Pap smear screening, specifically because women are culturally conditioned to establish family responsibilities such as children care and domestic work as priorities above their self-care, which may reduce their use of preventive care (26).

This study is consistent with and builds on the recommendation provided by Santamaría-Ulloa and Valverde-Manzanares (24) for Costa Rica by analyzing the reasons why women do not participate in Pap smear screening programs. We found that, as compared to not having an active sexual life, the odds of never having a Pap smear because of health care access issues or because of cultural reasons, increased with every year increment in age. Improving access to the health care system and understanding the factors that determine women’s health-related decision making is an important first step in understanding women’s motivations to have or not to have a vaginal cytology. Moreno and Rosales-Nieto (34) mention two existing factors that influence individuals’ health decision making: (1) the value an individual assigns to a specific goal and (2) his own perception regarding the probability that a specific action would contribute to attaining that goal. Access to vaginal cytology would therefore be mediated by a set of internal beliefs and evaluations that women make around how Pap smear actually contributes to attaining an optimal health status. In addition to identifying individual factors that shape health practices, it is also relevant to analyze and to intervene on those factors that explain how inequities affect Pap smear screening use. These inequities refer, for example, to educational attainment, health care access and the economic barriers identified in this study, which determine the outcomes of cervical cancer prevention strategies. It is not only necessary, but also urgent, to attain complete participation of women in the health care system by means of giving real opportunities according to their sexual and reproductive life specific needs.

## Conclusion

This study’s findings can be leveraged as evidence to* *inform public policy. With the goal of eradicating cervical cancer, it is important to address the structural barriers to self-care, promote actions that incentivize women’s self-care, and address the education needs of women about the importance of regular Pap smear screening. These will help women gain access to health care. Findings from this study are an input for public policies that focus on higher risk populations on which health care access barriers can be contained and diminished.

For those countries such as Costa Rica, that have already implemented HPV vaccination among girls, it is highly recommended to establish a surveillance system that allows the follow-up of vaccinated female patients as part of the national screening program. HPV molecular detection tests would also be an important affirmative action to eradicate cervical cancer on developing countries. Evidence- based decision making on sexual and reproductive health policy and planning are highly relevant to redirect resources and efforts to target the most vulnerable female population groups.

## Data Availability Statement

The raw data supporting the conclusions of this article may be requested through the following website of the Costa Rican Bureau of Statistics: https://www.inec.cr/encuestas/encuesta-nacional-de-hogares.

## Ethics Statement

Ethical review and approval was not required for the study on human participants in accordance with the local legislation and institutional requirements. Written informed consent for participation was not required for this study in accordance with the national legislation and the institutional requirements.

## Author Contributions

CS-U: Study design, data analysis, and manuscript writing. IQ-R: Study design and manuscript writing. MM-L: Data analysis and manuscript writing. HQ-L: Data analysis and manuscript writing. All authors contributed to the article and approved the submitted version.

## Conflict of Interest

The authors declare that the research was conducted in the absence of any commercial or financial relationships that could be construed as a potential conflict of interest.
